# YouTube™ as a source of information on food poisoning

**DOI:** 10.1186/s12889-019-7297-9

**Published:** 2019-07-16

**Authors:** Meng Li, Shoumeng Yan, Di Yang, Bo Li, Weiwei Cui

**Affiliations:** 1 Department of Epidemiology and Biostatistics, School of Public Health, Jilin University, 1163 Xinmin Avenue, Changchun, 130021 People’s Republic of China; 2 Changchun International Travel Healthcare, Changchun, 130061 People’s Republic of China; 3 Department of Nutrition and Food Hygiene, School of Public Health, Jilin University, 1163 Xinmin Avenue, Changchun, 130021 People’s Republic of China

**Keywords:** Food poisoning, Foodborne diseases, Foodborne illness, Epidemiology

## Abstract

**Background:**

YouTube™ (http://www.youtube.com), as a very popular video site around the world, is increasingly being used for health information. The objectives of this review were to assess the overall usefulness of information on food poisoning presented on YouTube™ for patients.

**Methods:**

The YouTube™ website was systematically searched using the key words “food poisoning”, “foodborne diseases” and “foodborne illness”. One hundred and sixty videos meet the inclusion criteria. Two independent reviewers scored the videos utilizing a customized usefulness scoring scheme separately and assessed the video duration, views, days since upload, likes, and dislikes. The videos were categorized as education, entertainment, News & Politics and People & Blogs. A usefulness score was devised to assess video quality and to categorize the videos into “slightly useful”, “useful”, and “very useful”.

**Results:**

Most videos were educational 66 (41.3%). Educational videos had significantly higher scores, but had no significant differences in likes, views or views/day. Over half of the videos (97/160) were categorized as “useful”. The mean posted days (885.2 ± 756.1 vs 1338.0 ± 887.0, *P* = 0.043) and the mean duration of video (12.8 ± 13.9 vs 3.5 ± 3.4, *P* < 0.001) were both significantly different in the very useful group compared with the slightly useful group. There was no correlation between usefulness and the number of likes, the number of dislikes, the number of views, or views/day.

**Conclusion:**

YouTube™ is a promising source of information regarding food poisoning. Educational videos are of highest usefulness. Considering that there is a lot of low-credibility information, consumers need to be guided to reliable videos in the field of healthcare information.

**Electronic supplementary material:**

The online version of this article (10.1186/s12889-019-7297-9) contains supplementary material, which is available to authorized users.

## Background

Food poisoning or foodborne diseases are usually infectious diseases caused by bacteria, fungi, viruses, parasites, or chemicals [1, 2]. The symptoms of foodborne diseases range from mild gastroenteritis to life-threatening syndromes. World Health Organization estimated the global and regional disease burden of 22 foodborne bacterial, protozoal, and viral diseases in 2010. And it is estimated that there were 582 million foodborne cases in 2010 [3]. Illness and death from foodborne disease are a constant threat to public health and socio-economic development all over the world [4]. A wide range of information including epidemiology, risk factor, etiology, symptom, treatment and prevention of foodborne diseases can be very useful for people to prevent and deal with food poisoning. These detailed information would provide people with a great awareness of risk and a improved perception to the real impacts of foodborne diseases.

People are increasingly using the Internet to access health information [5–7]. And it is quite convenient for people to obtain a wide array of health information. YouTube™ (http://www.youtube.com) represents one of the most popular social network sites for sharing video content behind Google [8]. YouTube™ was increasingly being used for health information [9], and it has a large community user base that allows users to view, upload, and download at free cost, and also communicate and comment easily between the sources of upload and other viewers [9]. However, using the Internet for health and medical information has a variety of disadvantages, including disorganization, complex medical language and lack of peer review [10]. Therefore it is essential to assess the quality of the information delivered [11–14]. To our knowledge, no prior studies have examined the availability of food poisoning education videos on YouTube™. Therefore, this study aims to systematically assess the usefulness of YouTube™ videos regarding food poisoning for improving the professionalism of video websites.

## Methods

### Search strategy

This study was exempt from Institutional Review Board approval of the study institution because it involved the use of public access data only. Using methods used previously [11], YouTube ™ (http://www.youtube.com) was systematically searched on May 10, 2018 for videos containing relevant information about food poisoning. In the Mesh (Medical Subject Headings, MeSH), it is explained that “foodborne disease” is often called “food poisoning”. In addition to “food poisoning”, other entry terms are combinations of “foodborne” and “disease/illness”. So, the search terms were determined as “food poisoning”, “foodborne disease” and “foodborne illness” to minimize the impact of other synonyms as much as possible. The search for these three terms yielded 697040 videos in total (food poisoning-673000 videos, foodborne disease-5740 videos, foodborne illness-18300 videos). The first 5 pages (20 videos/page × 5 pages = 100 videos) of each search result were screened on the assumption that users would not go beyond the first 5 pages of a search result [5, 15]. We used YouTube'’s default sorting option -- "“relevance"”, which may be the most commonly used option in the algorithms for YouTube sorting (relevance, upload date, views count, rating). All the advertisements in the search results and in the beginning of video were ignored.

The videos meet the inclusion criteria if they were:(1) in English; (2) available on May 10, 2018; (3) related to food poisoning in content. The exclusion criteria were: (1) videos that were not in English; (2) videos without sound; (3) advertisements; (4) animation or movie clip; (5) videos that did not relate to food poisoning such as music, gaming or others; (6) duplicate videos, in part or as a whole. In addition, videos with multiple series or parts were counted as one.

All the videos meeting the inclusion criteria were saved, and a document with uniform resource locators (URLs) and titles to all the selected videos was saved for backup. And all the preliminary searches were completed by two members of our investigative team separately for reliability and comparison.

### Video evaluation

We extracted information on the title, length of the video (in minutes), total views, days since upload, likes and dislikes. Calculations of views/day were performed. The videos was further divided into four categories [16, 17]: Education; Entertainment; News & Politics; People & Blogs. More specifically, medical courses or other academic videos were divided into “Education”; comedies and talk shows were divided into “Entertainment”; videos form government agencies and news reports about food poisoning incidents or outbreaks were divided into “News & Politics”; videos depicting personal food poisoning experiences or videos showing personal opinions about food poisoning were divided into “personal & blogs”. For example, a video describing the difference between a stomach flu and food poisoning (https://www.youtube.com/watch?v=EC7UaLIAEP4) was divided into “Education”.

A customized usefulness scoring scheme was created where scores were given for video quality and specific content. The Global Quality Scale (GQS) [18] was used to assess the overall quality of all selected videos. As shown in Table 1, GQS was a five-point Likert scale based on the quality of information, the flow and ease of use of the information present online. Seven content-specific items were also developed to assess whether videos discussed risk factors, epidemiology, etiology, symptoms, diagnosis, treatment and prevention of food poisoning. According to the video details of each item, the video was given 0 point (Not mentioned), 1 point (Briefly introduced) and 2 points (Introduced in detail). The total usefulness score is the sum of the GQS score and content score. According to the total score, the videos was categorized as slightly useful (1–6), useful (7–13), very useful (14–19).

**Table 1:** Global Quality Scale Criteria Used to Score Videos Containing Information About Food Poisoning

Score	Global Score Description
1	Poor quality, poor flow of the site, most information missing, not at all useful for patients
2	Generally poor quality and poor flow, some information listed but many important topics missing, of very limited use to patients
3	Moderate quality, suboptimal flow, some important information is adequately discussed but others poorly discussed, somewhat useful for patients
4	Good quality and generally good flow, most of the relevant information is listed, but some topics not covered, useful for patients
5	Excellent quality and excellent flow, very useful for patients

Each video was scored by two independent viewers (M. Li, S.M. Yan) who were knowledgeable in the risk factors, epidemiology, etiology, symptoms, diagnosis, treatment and prevention of food poisoning. If the content scores or GQS scores given by two viewers differ by three or more points, then the final score is given by an arbitrator (W.W. Cui). In addition to the scores given by the arbitrator, the scores given by the two viewers were then averaged to give an overall score that was used for final results and statistical analysis.

### Statistical analysis

SPSS 24.0 was used for data entry and analysis. The interclass correlation coefficient (ICC) was calculated to determine the degree of agreement between the two raters (Model: Two-Way Mixed; Type: Consistency). Cohen’s kappa coefficient (κ) [19] was also calculated to measure inter-rater agreement for the raters’ scores. Categorical variables were presented with frequency and percentage. Non-normally distributed continuous variables were represented with quartiles (25th percentile, 50th percentile and 75th percentile). Wilcoxon signed ranks test was used to test the difference between two scores given by the two viewers. Kruskal-Wallis test was used to determine whether differences existed between total usefulness score and category. A two-tailed *p*-value < 0.05 was deemed statistically significant.

## Results

**Fig. 1:**
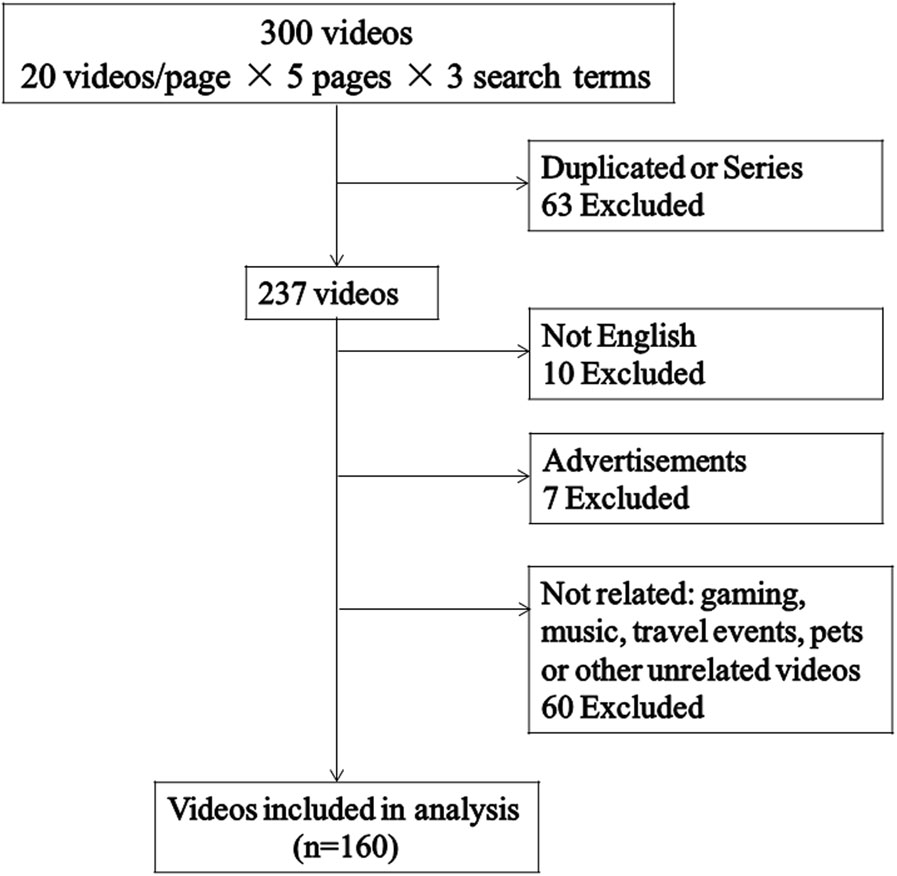
Details of videos included in the study

As shown in Fig. 1, 100 videos were screened for each of the three search terms (“food poisoning”, “foodborne disease”, “foodborne illness”), 160 unique videos met the inclusion criteria (total views, 8211201; total duration, 1194 min). An overview of the included videos is shown in Table 2. The majority of videos were educational (*n* = 66, 41.3%), with “People & Blogs” (*n* = 47, 29.4%) being the second most common category. The median duration of the videos was 4.0 min. Mean GQS score and content score of the videos was 3.8 ± 1.1, 5.8 ± 2.7 respectively. Mean total score of the videos included in the study was 9.7 ± 3.7.

**Table 2:** Summary of included YouTube™ videos about food poisoning

Characteristics	Total (*n* = 160)
Category n (%)	
Education	66 (41.3%)
Entertainment	12 (7.5%)
News & Politics	35 (21.9%)
People & Blogs	47 (29.3%)
Posted days	1146.5 (557.5,1846.0)
Duration	4.0 (2.0,8.0)
Views	3782.0 (739.8,14840.3)
Views/day	4.0 (0.5,16.8)
Likes	9.5 (1.0,89.0)
Dislikes	1.0 (0.0,7.0)
GQS score	4.0 (3.0,5.0)
Content score	6.0 (4.0,8.0)
Total score	10.0 (7.0,12.5)

Before the arbitrator intervened, there was no difference between content scores or GQS scores given by the two viewers (content score: z = − 1.500, *P* = 0.134; GQS score: z = − 1.414, *P* = 0.157;). As shown in Table 3, the final score using the customized scoring scheme demonstrated good reliability. The interclass correlation coefficient and Cohen’s kappa coefficient were calculated for GQS score, content score and total score respectively.

**Table 3:** Inter-rater reliability for customized usefulness scoring scheme

Reliability parameter	GQS score	Content score	Total score
ICC	0.978	0.995	0.995
κ	0.847	0.825	0.709

The classification of videos according to category with details of other characteristics is given in Table 4. There was no significant difference between category and likes, views or views/day (*P* = 0.106, *P* = 0.464, *P* = 0.113). Video duration and dislikes of the video were associated with category. Compared with News & Politics, People & Blogs videos were more likely to have a longer duration (*P* = 0.044). Entertainment videos had significantly more dislikes (*P* = 0.049). Since some videos had zero dislikes, likes/dislikes was not calculated. In addition, education videos were found to share over half the total duration, while People & Blogs videos share over half the total views. Results of Kruskal-Wallis test showed that GQS score and total score assessed by our reviewers correlated with category (*P* = 0.041 and *P* = 0.036 respectively). No significant correlation existed between content score and video category (*P* = 0.060).

**Table 4:** Detailed characteristics of videos based on category (median [quartile range])

	Education (*n* = 66)	Entertainment (*n* = 12)	News & Politics (*n* = 35)	People & Blogs (*n* = 47)	H	*P*
Likes	8.5 (1.0,28.0)	30.0 (4.3,263.8)	4.0 (1.0,47.0)	26.0 (1.0,192.0)	6.115	0.106
Dislikes	0.0 (0.0,4.3)	3.5 (0.0,15.8)	0.0 (0.0,4.0)	3.0 (0.0,14.0)	7.875	0.049^*^
Posted days	1480.0 (702.8,2034.5)	1095.0 (447.3,1682.5)	1428.0 (855.0,2101.0)	794.0 (340.0,1267.0)	10.780	0.013^*^
Duration (minutes)	4.0 (2.0,11.0)	4.5 (2.0,8.0)	3.0 (2.0,4.0)	5.0 (2.0,9.0)	8.100	0.044^*^
Views	2392.5 (720.5,10827.3)	9290.0 (1118.0,23044.8)	2055.0 (654.0,16095.0)	6319.0 (781.0,28296.0)	2.564	0.464
Views/day	2.4 (0.6,9.3)	7.2 (1.2,26.6)	2.0 (0.3,13.4)	12.0 (0.9,31.4)	5.981	0.113
Total duration (minutes [%])	661 (55.36%)	57 (4.77%)	190 (15.91%)	286 (23.95%)	–	–
Total views (n [%])	1237876 (15.08%)	312444 (3.81%)	2316474 (28.21%)	4344407 (52.91%)	–	–
GQS score	4.5 (3.0,5.0)	3.0 (2.3,4.4)	3.0 (3.0,5.0)	4.0 (3.0,5.0)	8.238	0.041^*^
Content score	6.3 (4.0,9.0)	4.5 (3.3,6.0)	5.0 (4.0,7.0)	5.0 (3.0,8.0)	7.422	0.060
Total score	10.8 (8.0,14.0)	7.5 (6.0,10.4)	9.0 (6.0,11.0)	9.0 (7.0,13.0)	8.568	0.036^*^

*statistically significant

The videos were further categorized into “slightly useful”, “useful” and “very useful” according to their total usefulness score. Three videos were determined to be most useful for food poisoning (see Additional file 1: Table S1). Video demographics according to usefulness are shown in Table 5. More than half of the videos were useful (*n* = 97, 60.6%), while 38 of the videos (23.8%) were slightly useful, and only 25 of the videos (15.6%) were deemed very useful.

**Table 5:** Detailed characteristics of videos based on usefulness (median [quartile range])

	Slightly useful (*n* = 38)	Useful (*n* = 97)	Very useful (*n* = 25)	H	*P*
Likes	5.0 (0.8,34.3)	10.0 (1.5,92.0)	14.0 (0.0,203.5)	1.451	0.484
Dislikes	0.5 (0.0,5.0)	1.0 (0.0,12.0)	0.0 (0.0,9.0)	1.968	0.374
Posted days	1158.0 (715.8,1844.5)	1378.0 (559.0,2019.0)	707.0 (275.0,1268.0)	6.300	0.043^*^
Duration (minutes)	2.0 (1.0,5.0)	4.0 (2.0,8.5)	7.0 (4.0,18.5)	21.248	< 0.001^*^
Views	2007.5 (684.8,10202.5)	5560.0 (975.5,19374.0)	1767.0 (228.5,39970.5)	3.348	0.187
Views/day	2.3 (0.3,13.2)	4.7 (0.7,20.4)	2.8 (0.4,43.5)	1.507	0.471
Total duration (minutes [%])	134 (11.22%)	739 (61.89%)	321 (26.88%)	–	–
Total views (n [%])	398259 (4.85%)	6886005 (83.86%)	926937 (11.29%)	–	–
Category				–	–
Education	14	35	17		
Entertainment	4	8	0		
News & Politics	9	24	2		
People & Blogs	11	30	6		

*statistically significant

There were no significant differences between groups regarding the number of likes, dislikes, views, or views per day. The number of posted days in the very useful group was 885.2 ± 756.1, which was significantly shorter than other groups (*P* = 0.043). The median duration in the very useful group was significantly longer than other groups (*P* < 0.001). Chi-square test showed no correlation between the usefulness category and video category (Fisher’s exact test, *P* = 0.118).

## Discussion

To the best of our knowledge, this study is the first to evaluate the information available on YouTube™ regarding food poisoning. We evaluated 160 videos with total cumulative duration of 19.9 h and viewership of over 8 million, provided a detailed analysis of the YouTube™ videos as a source of medical information. Overall, the included videos were mixed in addressing the specific content of food poisoning. Not surprisingly, symptoms, treatment and prevention of food poisoning were mostly discussed, epidemiology and diagnosis of food poisoning were less discussed. Out of the 160 videos, 97(60.6%) videos were useful, and a minority (23.75%) were slightly useful, showing that YouTube™ was a important tool for useful information broadcasting. The percentage of useful videos is higher compared with that of previous studies evaluating video content on other topics [17, 20–22]. This may be due to the great harm of food poisoning, so it was paid more attention.

Among the 160 videos included in our study, 47 (29.3%) were “People & Blogs” videos. These videos are almost personal experience or opinions on food poisoning. This indicated that individuals not only use the Internet to seek health information, but also use the Internet to spread health and medical information. Not unexpectedly, educational videos had a significant higher score and less dislikes than “People & Blogs” videos, and this was similar to previous study assessing information of YouTube™ videos dealing with Type 2 Diabetes [23]. Educational videos usually involved the symptoms, etiology, treatment and prevention of food poisoning, while “People & Blogs” videos often involved symptoms only. Furthermore, “People & Blogs” videos were viewed more than education videos, possibly because viewers find this kind of video more interesting, or these videos were uploaded by some social celebrities they followed. No significant difference was found in the views or the likes across the categories, and this may be because our search results are limited in the first five pages of the videos in accordance with the “relevance” order.

After videos being categorized as slightly useful, useful and very useful, we found that “very useful” videos had the longest duration, and were uploaded more recently. This is different from the previous study focused on mouth (oral) cancer [24], which showed no significant correlation between the usefulness score and video length. This may be because educational videos account for a different percentage of the total video (152/188 vs. 66/160). However, it was concerning to find that there was no difference in the “likes”, “dislikes”, and “views” between videos of different levels of usefulness, suggesting that the users probably can not judge the quality of information presented on YouTube™. Consequently, “likes”, “dislikes”, and “views” cannot be used to help identify the usefulness of videos, which is consist with previous systematic review of information on YouTube™ regarding femoroacetabular impingement [25].

Worldwide foodborne diseases are an important cause of morbidity, mortality, and economic costs [26]. Since the incubation period for food poisoning is usually short, the patients have sudden onset and their conditions progress rapidly. If the patients cannot be diagnosed and treated in time, the consequences will be more serious. Therefore, videos disseminating correct and critical information about food poisoning are of great significance. However, as listed in (see Additional file 1 Table S1), the number of views for the top three most useful videos identified by our scoring scheme was relatively low. The three videos were uploaded in 2016, 2014 and 2012 respectively. They all introduced risk factors, epidemiology, etiology and prevention of food poisoning in detail. The reason for their low views maybe that they are highly professional and they have longer video durations. Therefore, proper duration and entertainment of the videos count a great deal in spreading health information.

This study has several limitations. First, it is subjective to evaluate the videos using the customized usefulness scoring scheme, as there are no validated tools for assessing video data yet. Second, the videos were sorted by relevance, which is the YouTube™ default. The searching results may be affected by sorting standard or advertisements. Third, YouTube™ search results are dynamic, and will change when new videos are uploaded or old videos are removed. So, this cross-sectional study demonstrates the usefulness of information on food poisoning at that time.

## Conclusions

In conclusion, a large amount of information on food poisoning is available on YouTube™. More than half of the YouTube™ videos regarding food poisoning are useful, and provide information of symptoms, treatment or prevention on food poisoning. Overall, educational videos were more useful regarding providing information on food poisoning. Considering that there is a lot of low-credibility information on the Internet, consumers need to be guided to reliable videos in the field of healthcare information.

## Additional file

Additional file 1: **Table S1** Most Useful Videos as Identified by Applying Scoring Scheme. (DOC 28 kb)

## Data Availability

The datasets used and/or analysed during the current study are available from the corresponding author on reasonable request.

## Abbreviations

GQS: Global Quality Scale
ICC: Interclass Correlation Coefficient
URL: Uniform Resource Locators
