# Supplementary material for: YouTube™ as a source of information on food poisoning
Source: BMC Public Health. 2019 Jul 16;19:952. doi: 10.1186/s12889-019-7297-9 (PMC6636170; doi:10.1186/s12889-019-7297-9)
Supplement: Supplementary file 1 — Table S1 Most Useful Videos as Identified by Applying Scoring Scheme. (DOC 28 kb) [file 12889_2019_7297_MOESM1_ESM.doc]

Table S1 Most Useful Videos As Identified by Applying Scoring Scheme

| Title | URL |
| --- | --- |
| The Impact of Foodborne Diseases Around the World: WHO Global Burden of Foodborne Disease | https://www.youtube.com/watch?v=DBmcROD-sqc |
| Food Poisoning Symptoms, Causes and Treatments | https://www.youtube.com/watch?v=4LHgSpZsT8A |
| Foodborne Pathogens -- Sandra Gompf, MD | https://www.youtube.com/watch?v=0AUmBiWiwNQ |
